# Psychiatric symptoms and necroinflammatory activity in chronic hepatitis B: a cross-sectional study

**DOI:** 10.1186/s12879-025-12033-8

**Published:** 2025-11-14

**Authors:** Nurbanu Sezak, Ozge Eren Korkmaz, Burcu Acikalin Arikan, Hasan T. Kilic, Pelin Soydar

**Affiliations:** 1 Department of Infectious Disease and Medical Microbiology, Buca Seyfi Demirsoy Education and Training Hospital, Izmir Demokrasi University School of Medicine, Izmir, Türkiye; 2 Department of Epidemiology, Faculty of Medicine, Dokuz Eylul University, Izmir, Türkiye; 3 Buca Seyfi Demirsoy Education and Training Hospital, Infectious Diseases Clinic, Izmir, Türkiye; 4 Department of Psychiatry, Faculty of Medicine, Buca Seyfi Demirsoy Education and Training Hospital, Izmir Demokrasi University, Izmir, Türkiye; 5 Department of Neurology, Ataturk State Hospital, Aydın, Türkiye

**Keywords:** Chronic hepatitis B, Depression, Anxiety, Fatigue, Liver fibrosis, Non-invasive fibrosis scores, Quality of life

## Abstract

**Background:**

Patients with chronic hepatitis B (CHB) are at risk of developing psychiatric symptoms such as depression, anxiety, and fatigue, which may adversely affect quality of life, treatment adherence, and disease outcomes. Emerging evidence suggests that these psychological symptoms are influenced not only by psychosocial stressors but also by biological mechanisms related to hepatic necroinflammation and fibrosis. This study aimed to investigate the relationship between depression, anxiety, fatigue, and non-invasive liver fibrosis scores in patients with CHB.

**Methods:**

In this cross-sectional study, 200 adult CHB patients were consecutively recruited from a university-affiliated hospital outpatient clinic between September 2023 and February 2024. Patients with acute HBV, cirrhosis, hepatocellular carcinoma, psychiatric comorbidities, or major systemic illnesses were excluded. Psychiatric symptoms were assessed using the validated versions of the Hospital Anxiety and Depression Scale (HADS) and the Fatigue Severity Scale (FSS). Liver fibrosis and necroinflammatory activity were evaluated using non-invasive markers, including AST/ALT ratio (AAR), APRI, FIB-4, and Age-Platelet Index (API). Multivariable logistic regression was used to identify independent predictors of depression, anxiety, and fatigue.

**Results:**

Of the 200 patients, 21.0% had depression, 6.5% had anxiety, and 27.0% reported significant fatigue. APRI values were significantly higher in patients with fatigue (*p* = 0.002), and API scores were significantly higher in those with anxiety (*p* = 0.035). No significant association was found between non-invasive fibrosis indices and depression, although patients with higher depression scores tended to have elevated AAR values. Anxiety was more frequent among unmarried individuals (*p* < 0.001), and fatigue was more common in patients receiving antiviral treatment (*p* = 0.027).

**Conclusions:**

Psychiatric symptoms are prevalent among CHB patients and show significant associations with both psychosocial and biological disease factors. Fatigue was associated with hepatic injury reflected by APRI, and anxiety with early fibrosis markers such as API. These findings underscore the need to integrate routine *psychiatric* screening for depression, anxiety, and fatigue into CHB management, and to address these symptoms through comprehensive, multidisciplinary care approaches to improve patient well-being and treatment adherence.

**Trial registration:**

Not applicable. This study is a cross-sectional observational design and did not require prospective registration.

## Background

Preserving physical, psychological, and social well-being is a challenge for individuals with chronic hepatitis B (CHB), akin to other chronic illnesses [1]. In recent years, there has been growing recognition of the importance of assessing not only physical symptoms but also health-related quality of life (HRQoL) in patients with CHB.

Psychiatric comorbidities such as depression and anxiety are increasingly reported in this population and have been shown to significantly impair HRQoL [2]. In recent years, studies have been conducted on the immunological and inflammatory mechanisms underlying the increased incidence of depression and anxiety in these patients [3–5]. Emerging research suggests a complex, bidirectional interaction between chronic inflammation and psychological symptoms: on one hand, persistent inflammation in CHB may contribute to the development of mood disorders; on the other hand, depression and anxiety may negatively influence disease progression via dysregulation of the immune system and poor treatment adherence.

This study aims to evaluate the relationship between liver necroinflammation/fibrosis and psychiatric symptoms — specifically anxiety, depression, and fatigue — among patients with chronic hepatitis B, using validated psychological scales and non-invasive clinical parameters.

## Materials and methods

### Study design

This was a cross-sectional observational study with consecutive sampling. Participants were consecutively recruited from the Infectious Diseases outpatient clinic of a university affiliated education and training hospital between *September 2023 and February 2024*. The study adhered to the ethical standards of the Declaration of Helsinki (1975, revised 2008) and was approved by the institutional ethics committee.

### Participants

**Fig. 1:**
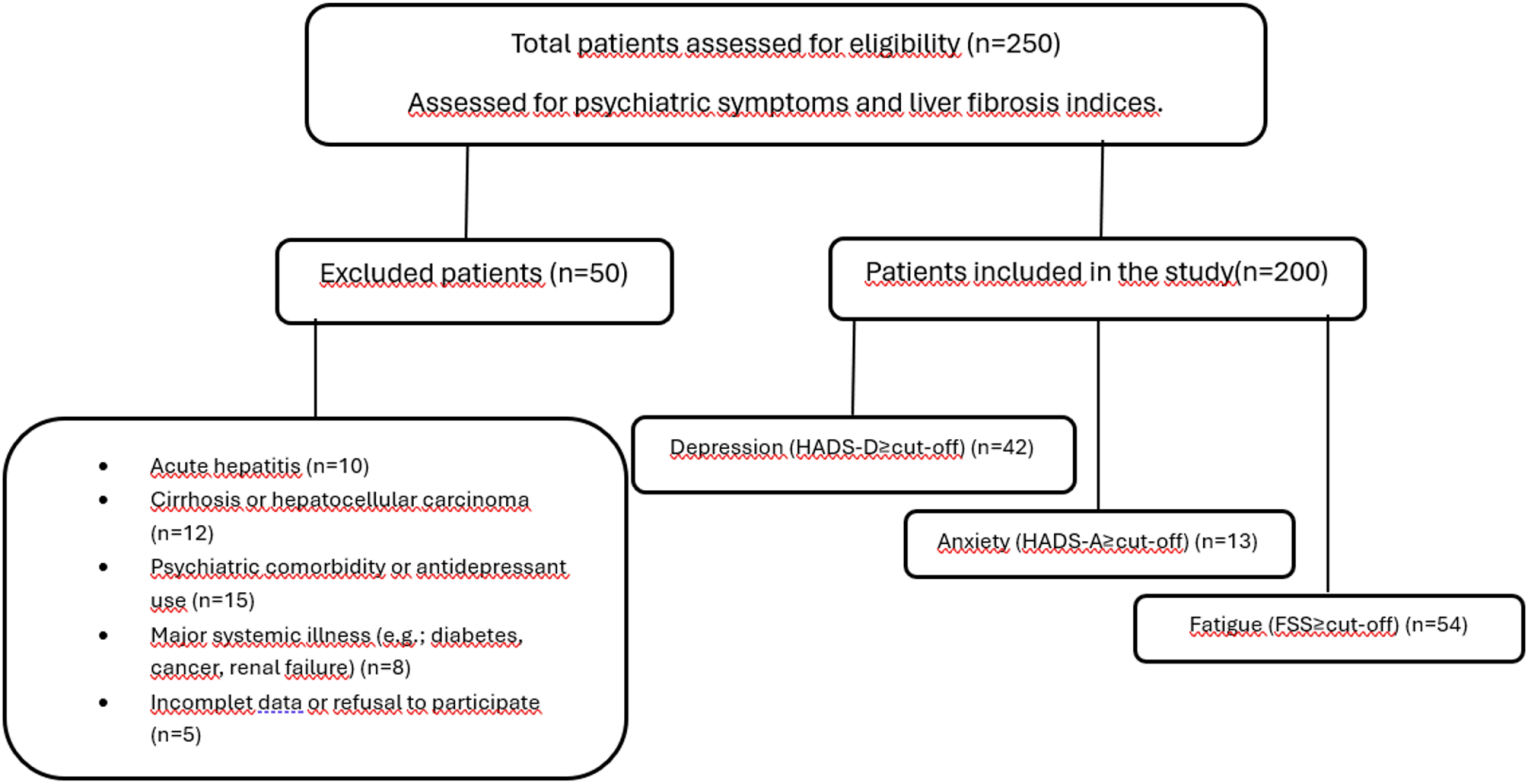
STROBE-compliant patient flow diagram

Eligible participants were adults (≥ 18 years) diagnosed with chronic hepatitis B (CHB) and managed in accordance with the European Association for the Study of the Liver (EASL) guidelines [6]. However, the decision to start treatment for patients was made in line with the reimbursement policy of our national Health Implementation Guideline (HIG), which applies stricter criteria than the EASL guidelines. Consequently, some patients with low-to-moderate HBV DNA levels and normal ALT values remained untreated. Patients with acute hepatitis B, other chronic liver diseases (hepatitis C or D coinfection, alcoholic or metabolic dysfunction-associated fatty liver disease, autoimmune hepatitis, drug-induced liver injury, cirrhosis, hepatocellular carcinoma), systemic diseases such as diabetes mellitus, chronic kidney disease, malignancy, and those with known psychiatric disorders or current antidepressant use were excluded from the study. Pregnant women, individuals with substance or alcohol abuse, and patients who were hospitalized for any reason during data collection were also excluded. Sociodemographic data including age, sex, and marital status were collected using structured forms based on patient interviews. The enrollment and exclusion process of the study population is illustrated in Fig. 1, which presents the STROBE-compliant patient flow diagram showing eligibility assessment, exclusion reasons, and final inclusion.

### Clinical and laboratory data

Biochemical parameters including Aspartate aminotransferase (AST) (IU/L), Alanine aminotransferase (ALT) (IU/L), platelet count ​​(10³/mm³), and serum HBV-DNA level (IU/mL), along with antiviral treatment regimens, were extracted from hospital records.

The non-invasive markers of liver fibrosis and necroinflammation were calculated:

- AST/ALT ratio (AAR) >1 indicated fibrosis [7];
- Fibrosis-4 index (FIB-4) ≤ 1.45 suggested absence of fibrosis, whereas ≥ 3.25 indicated advanced fibrosis [8];
- Age-platelet Index (API) ≥ 6 indicated fibrosis [9, 10];
- AST/Platelet ratio (APRI score) cut-off value of 1.0 was used to indicate advanced fibrosis (F3–F4 equivalent), whereas values < 1.0 were considered consistent with non-advanced fibrosis (F1–F2 equivalent), according to established literature [7–9].

Patients were considered to have fibrosis if any one of these markers met the respective fibrosis threshold.

### Psychological assessments

Psychological symptoms were assessed using validated Turkish versions of.

- Hospital Anxiety and Depression Scale (HADS) for anxiety and depression [11],
- Fatigue Severity Scale (FSS) for fatigue assessment [12].

Clinically significant symptoms were defined as scores ≥ 8 on each HADS subscale and mean FSS ≥ 4.

### Statistical analysis

All analyses were conducted in R (v2024.04.1 + 748). The normality assumption was assessed using the Shapiro–Wilk test. Continuous variables with a normal distribution were summarized as mean ± standard deviation (SD), while non-normally distributed variables were presented as median [Q1–Q3]. Categorical variables were expressed as frequency and percentage (%). Comparisons between categorical variables were performed using the Chi-square test or Fisher’s Exact test, whereas for continuous variables, the independent samples t-test was applied to normally distributed data and the Mann–Whitney U test to non-normally distributed data.

The expected frequency of 40 ± 5% referred to the estimated prevalence of depressive symptoms among patients with chronic hepatitis B, based on data from previous studies in similar clinical populations [13, 14]. We specified an absolute precision (d) of 5%, confidence level of 80% and design effect (DEFF) = 1 given single-center consecutive recruitment (no clustering). The minimum sample size was *n* = 158. A total of 200 participants were prospectively enrolled to ensure sufficient statistical power and allow for subgroup and multivariable regression analyses.

Multivariable logistic regression with backward stepwise selection was used to identify independent predictors of depression, anxiety, and fatigue. Multicollinearity was checked using the Variance Inflation Factor (VIF); variables with VIF < 5 were retained. Model fit was assessed using the Hosmer–Lemeshow goodness-of-fit-test. Analyses used “dplyr”, “stats”, “MatchIt”, “MASS”, “car”, and “ggplot2” packages. A p-value < 0.05 was considered statistically significant.

### Ethical considerations

The study protocol was reviewed and approved by the Ethics Committee of Buca Seyfi Demirsoy Training and Research Hospital with decision number 2023/8-160. Written informed consent was obtained from all participants prior to enrollment. Participants who scored above the screening thresholds for depression or anxiety were informed about their results and referred to the psychiatry outpatient clinic for further evaluation, in accordance with the principles of the Declaration of Helsinki.

## Results

A total of 200 patients with chronic hepatitis B (CHB) were included in the study. The median age was 49.5 years (Q1-Q3: 39.8–59.2), and 52.5% were female. The majority (90.5%) were married and the median duration of HBV infection was 14.0 years (Q1-Q3:10.0–20.0). Just over half (52.5%) were not receiving antiviral therapy at the time of assessment.

According to psychological assessments, 21.0% of patients had depression, 6.5% had anxiety, and 27.0% reported significant fatigue based on the applied scales.

Patients with depression were significantly younger than those without (48.0 vs. 51.0 years; *p* = 0.046) and also had a shorter duration of HBV infection (median 13.5 vs. 15.0 years; *p* = 0.022).

Anxiety was more common in women (10.5%) than men (2.9%), with a borderline significant difference (*p* = 0.056). Marital status was strongly associated with anxiety; unmarried patients exhibited a markedly higher prevalence (31.6%) compared with married individuals (3.9%, *p* < 0.001).

Fatigue was more prevalent in patients receiving antiviral therapy (34.7% vs. 20.0%, *p* = 0.029). Additionally, those reporting fatigue had significantly lower serum HBV-DNA levels (median 0.00 IU/mL vs. 85.0 IU/mL, *p* = 0.036 was associated with lower HBV-DNA levels (median 0.00 IU/mL vs. 85.0 IU/mL, *p* = 0.036). No significant differences in HBV-DNA levels were observed between patients with or without anxiety or depression. Table 1 provides a detailed comparison of demographic and clinical characteristics according to depression, anxiety, and fatigue status.

**Table 1:** Comparison of demographic and clinical characteristics of patients based on the HADS and FSS results

Characteristics	Total	Depression			Anxiety			Fatigue		
	*N*=200	Have *n*=42 (21.0%)	None *n*=158 (79.0%)	*P*	Have *n*=13 (6.5%)	None *n*=187 (93.5%)	*P*	Have *n*=54 (27.0%)	None *n*=146 (73.0%)	*P*
Age, years, median [Q1-Q3]	49.5 [39.8-59.2]	48.0 [38.5-55.0]	51.0 [40.0-60.0]	.**046**	52.0 [38.0-55.0]	49.0 [40.0-60.0]	0.732	50.0 [42.0-60.0]	49.0 [37.0-58.8]	0.341
Sex, n (%)				0.394			**0.056**			0.555
Male	105 (52.5%)	25 (23.8%)	80 (76.2%)		3 (2.9%)	102 (97.1%)		26 (24.8%)	79 (75.2%)	
Female	95 (47.5%)	17 (17.9%)	78 (82.1%)		10 (10.5%)	85 (89.5%)		28 (29.5%)	67 (70.5%)	
Marital status, n (%)				0.083			**<0.001**			0.841
Single	19 (9.5%)	7 (36.8%)	12 (63.2%)		6 (31.6%)	13 (68.4%)		6 (31.6%)	13 (68.4%)	
Married	181 (90.5%)	35 (19.3%)	146 (80.7%)		7 (3.9%)	174 (96.1%)		48 (26.5%)	133 (73.5%)	
Duration of HBV infection, years median [Q1-Q3]	14.0 [10.0-20.0]	13.5 [6.50-17.2]	15.0 [10.0-20.0]	**0.022**	18.0 [6.00-20.0]	14.0 [10.0-20.0]	0.940	14.0 [10.0-17.8]	15.0 [9.25-20.0]	0.792
Antiviral treatment n (%)				0.297			0.267			**0.008**
None	105 (52.5%)	19 (18.1%)	86 (81.9%)		7 (6.7%)	98 (93.3%)		21 (20.0%)	84 (80.0%)	
TDF	60 (30.0%)	17 (28.3%)	43 (71.7%)		2 (3.3%)	58 (96.7%)		17 (28.3%)	43 (71.1%)	
Entecavir	24 (12.0%)	6 (25.0%)	18 (75.0%)		4 (16.7%)	20 (83.3%)		9 (37.5%)	15 (62.5%)	
Lamivudine	5 (2.5%)	0 (0.0%)	5 (100.%)		0 (0.0%)	5 (100%)		2 (40.0%)	3 (60.0%)	
TAF	6 (3.0%)	0 (0.0%)	6 (100%)		0 (0.0%)	6 (100%)		5 (83.3%)	1 (16.7%)	
Use antiviral treatment, n (%)				0.375			1.00			**0.029**
Have	95 (47.5%)	23 (24.20)	72 (75.8%)		6 (6.3%)	89 (93.7%)		33 (34.7%)	62 (65.3%)	
None	105 (52.5%)	19 (18.10)	86 (81.9%)		7 (6.7%)	98 (93.3%)		21 (20.0%)	84 (80.0%)	
HBV-DNA (IU/mL) median [Q1-Q3]	40.0 [0.00-700]	15.0 [0.00-1000]	55.0 [0.00-600]	0.582	10.0 [0.00-700]	50.0 [0.00-700]	0.902	0.00 [0.00-465]	85.0 [0.00-795]	**0.036**
HBV-DNA (IU/mL), mean ±SD	142,946±1,646,520	549,037±3,548,754	34,998 ±302,721	0.354	990 ± 2732	152,815 ±1,702,646	0.224	426,434 ±3,129,833	38,095 ±314,801	0.367
Fibrosis				0.355			0.318			0.893
Have	104 (52.0%)	25 (24.0%)	79 (76.0%)		9 (8.7%)	95 (91.3%)		29 (27.9%)	75 (72.1%)	
None	96 (48.0%)	17 (17.7%)	79 (82.3%)		4 (4.2%)	92 (95.8%)		25 (26.0%)	71 (74.0%)	
AAR median [Q1-Q3]	1.00 [0.80-1.20]	1.15 [0.86-1.26]	1.00 [0.80-1.20]	0.343	1.09 [0.86-1.28]	1.00 [0.80-1.20]	0.360	1.06 [0.86-1.20]	1.00 [0.80-1.23]	0.320
FIB-4 median [Q1-Q3]	0.90 [0.67-1.39]	1.00 [0.66-1.31]	0.88 [0.67-1.39]	0.842	0.86 [0.68-0.99]	0.91 [0.66-1.39]	0.559	1.00 [0.72-1.44]	0.88 [0.65-1.31]	0.156
API median [Q1-Q3]	3.00 [2.00-5.00]	3.00 [2.00-5.00]	3.00 [2.00-4.00]	0.821	3.00 [2.00-5.00]	3.00 [1.00-3.00]	**0.035**	3.00 [2.00-4.00]	3.00 [2.00-5.00]	0.230
APRI median [Q1-Q3]	0.20 [0.20-0.30]	0.20 [0.12-0.30]	0.20 [0.20-0.30]	0.670	0.20 [0.10-0.20]	0.20 [0.20-0.30]	0.132	0.20 [0.20-0.30]	0.20 [0.10-0.20]	**0.002**

SD: Standard Deviation, HADS: Hospital Anxiety and Depression Scale, FSS: Fatigue Severity Score, HBV: Hepatitis B Virus, TDF: tenofovir disoproxil fumarate, TAF: tenofovir alafenamide., AAR: AST/ALT ratio, FIB-4: Fibrosis-4 Score, API: Age-Platelet Index, APRI: AST/Platelet ratio

APRI values were significantly higher in patients with fatigue compared to those without fatigue (*p* = 0.002). API scores were also significantly higher in patients with anxiety than in those without anxiety (*p* = 0.035). AAR and FIB-4 scores did not show any significant differences between the depression, anxiety, and fatigue groups (*p* > 0.05) (Table 1).

**Fig. 2:**
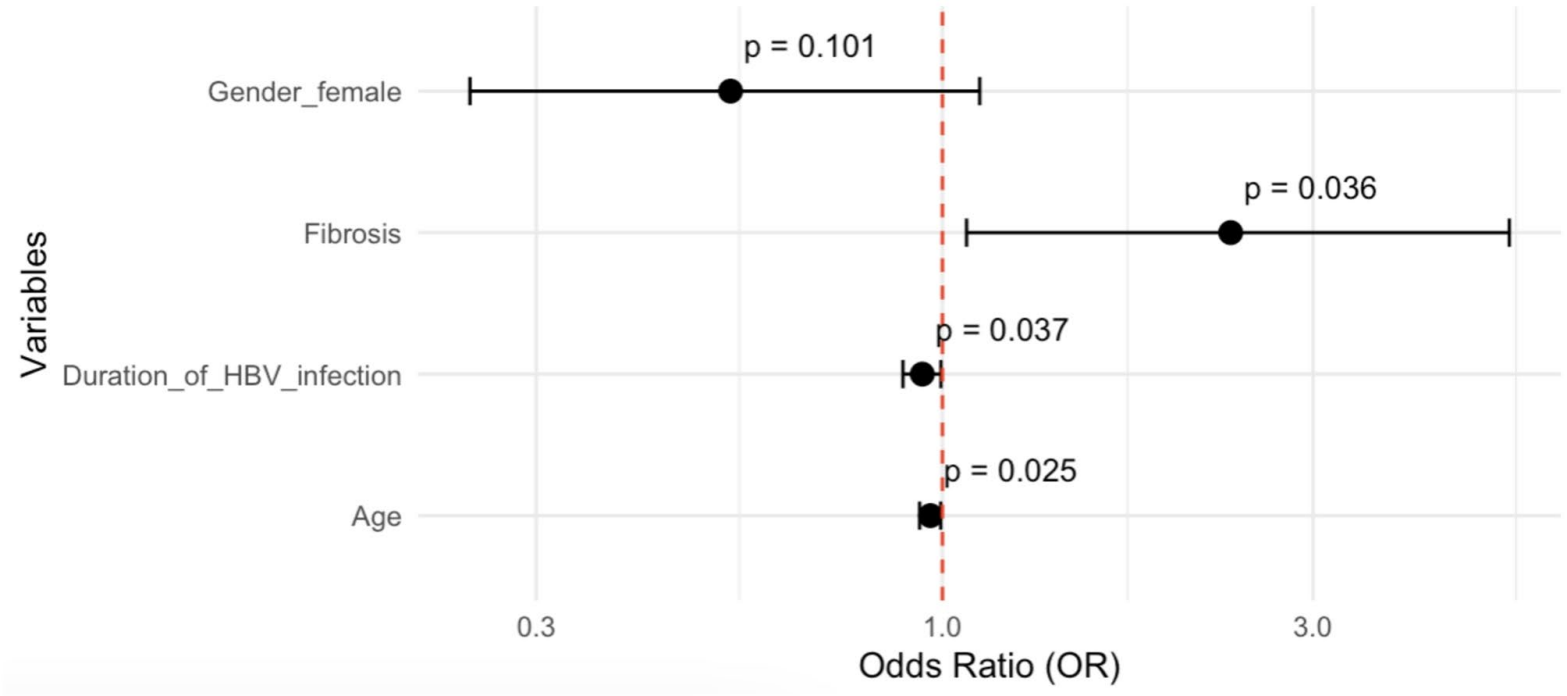
Depression risk factors: Odds ratios and 95% confidence intervals from multivariable stepwise backward model (Hosmer and Lemeshow p-value = 0.060)

**Fig. 3:**
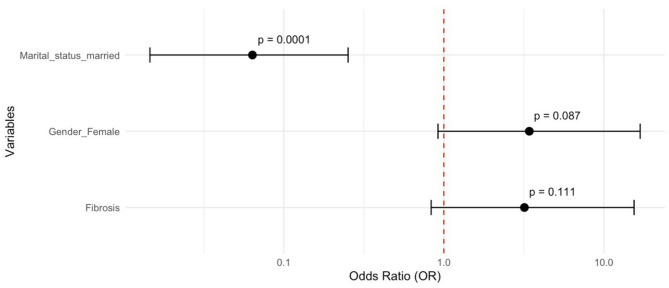
Anxiety risk factors: Odds ratios and 95% confidence intervals from multivariable stepwise backward model (Hosmer and Lemeshow p-value = 0.8522)

**Fig. 4:**
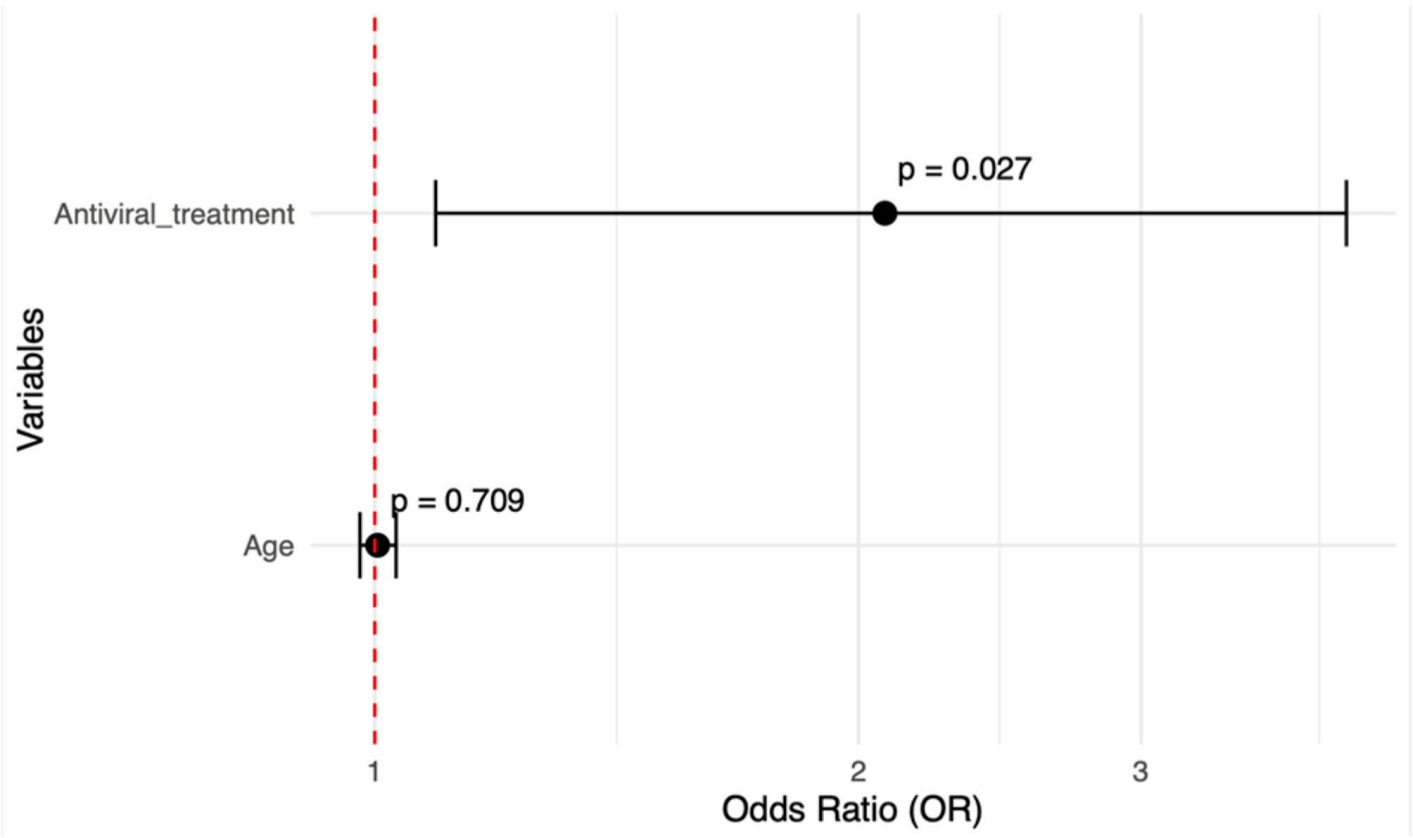
Fatigue severity risk factors: Odds ratios and 95% confidence intervals from multivariable stepwise backward model (Hosmer-Lemeshow p-value = 0.839)

Multivariable logistic regression models identified the following independent predictors. **Depression** was significantly associated with liver fibrosis (OR = 2.35, 95% CI: 1.07–5.36; *p* = 0.036). Increasing age (OR = 0.96 per year, *p* = 0.025) and longer infection duration (OR = 0.94 per year, *p* = 0.037) were protective factors (Fig. 2). **Anxiety** was more likely in females (OR = 3.42), though this did not reach statistical significance (*p* = 0.087). Being married was strongly protective (OR = 0.06, 95% CI: [0.01, 0.25], p = < 0.001), corresponding to a 94% lower likelihood of anxiety (Fig. 3). **Fatigue** was significantly associated with antiviral treatment (OR = 2.07, 95% CI: 1.09–4.02, *p* = 0.027), suggesting that treatment status may influence fatigue perception (Fig. 4). Figures 2, 3 and 4 visualize the odds ratios and 95% confidence intervals for key predictors identified in each model.

## Discussion

This study investigated the relationship between psychological symptoms—namely depression, anxiety, and fatigue—and liver necroinflammatory activity in patients with chronic hepatitis B (CHB). Our findings demonstrated significant associations between non-invasive fibrosis markers and these psychological outcomes, underscoring the complex interaction between mental health and liver disease severity. In the univariate analyses, APRI values were significantly higher in patients with fatigue compared to those without fatigue (*p* = 0.002). API scores were also significantly higher among patients with anxiety than in those without anxiety (*p* = 0.035). In contrast, AAR and FIB-4 scores showed no significant differences between groups with and without depression, anxiety, or fatigue (*p* > 0.05). These results highlight the importance of considering psychosocial dimensions when managing patients with CHB.

Chronic hepatitis B infection can lead to severe hepatic complications, including liver cirrhosis, hepatic failure, and hepatocellular carcinoma. These outcomes arise from persistent viral replication and immune-mediated hepatocellular injury that promote necroinflammatory activity and fibrotic progression [15, 16]. Such complications are not only life-threatening but also impose substantial psychological distress, often contributing to depression, anxiety, and fatigue due to uncertainty about disease prognosis and the burden of lifelong management. Our findings of a link between psychiatric symptoms and necroinflammatory markers may thus represent an early reflection of the psychosomatic consequences of progressive liver disease.

Although the difference did not reach statistical significance, patients with higher depression scores tended to have elevated AAR values. This trend may suggest a potential link between hepatic functional imbalance and depressive symptoms, which warrants further investigation in larger cohorts. Similar findings have been reported in previous studies showing that depression in chronic HBV-infected patients may be associated with immune dysregulation and increased inflammatory cytokines, such as IL-6, IL-8, TNF-α, and TGF-β [4, 17]. Cho et al. further showed that depressive symptoms in HBV-infected individuals increased the risk of liver-related mortality, underlining the clinical importance of this association [18]. Interestingly, we observed that older age and longer duration of HBV infection were protective against depression. This finding contrasts with studies reporting an increased prevalence of depression in older CHB patients [19], and may reflect improved coping strategies and greater psychological adaptation over time.

Anxiety in our cohort was more frequent among women and unmarried individuals. While the gender effect did not reach statistical significance in multivariable analysis, it aligns with prior studies highlighting female sex as a risk factor for psychiatric morbidity in chronic viral hepatitis [19, 20]. The strong protective effect of marriage observed in our study supports the role of social support systems in mitigating psychological distress, consistent with evidence linking marital status to better quality of life in liver disease [21, 22]. Beyond psychosocial aspects, patients with anxiety also showed significantly higher API scores compared with those without anxiety (*p* = 0.035). This finding may indicate a potential association between early hepatic architectural changes and anxiety symptoms, possibly mediated through systemic inflammatory or metabolic pathways. Bakhshi et al. showed that anxiety was associated with up-regulation of CD36 in monocytes of CHB patients [23], while Bahramabadi et al. demonstrated altered cytokine profiles (IL-6, IL-8, TNF-α, TGF-β) in those with psychiatric symptoms [17], supporting a biological link between inflammation and anxiety.

Fatigue emerged as another critical symptom, affecting over one-quarter of participants. Its association with APRI scores (*p* = 0.002) suggests a possible relationship between hepatic injury and fatigue severity, consistent with previous findings from large cohort studies [24, 25]. Moreover, antiviral treatment was significantly associated with greater fatigue, a finding that diverges from some reports in the literature [26]. This may reflect challenges related to long-term medication adherence and treatment-related side effects, rather than direct antiviral toxicity. This discrepancy may reflect challenges of long-term medication adherence, a point emphasized by Kurt et al., showed that drug compliance in CHB patients is closely tied to psychological burden [27]. These results indicate that even mild hepatic injury reflected by higher APRI values may contribute to fatigue, possibly through systemic inflammatory or metabolic mechanisms. Given the clinical significance of fatigue for patient functionality and well-being, these results reinforce the need to address this symptom as part of comprehensive CHB management.

Our study also adds to the ongoing debate regarding the psychiatric effects of antiviral therapy. While interferon therapy has long been associated with depression, the psychiatric impact of modern oral antivirals remains unclear. Some studies have reported that psychiatric symptoms persisted or increased in patients despite antiviral treatment [28]. Consistent with some prior work [2], we did not observe a significant relationship between antiviral use and depression or anxiety; however, the observed increase in fatigue among treated patients suggests that biochemical efficacy does not always translate into improved psychological outcomes. Clinicians should remain attentive to these aspects when monitoring patients on long-term antiviral therapy.

Given that our study demonstrated a relationship between psychiatric symptoms and necroinflammatory activity in patients with chronic hepatitis B, it is also important to consider how other viral infections might influence similar pathways. Recent data have highlighted the long-term neuropsychiatric and inflammatory consequences of COVID-19 infection. Guo et al. reported that post-COVID autoimmunity and persistent systemic inflammation may contribute to neurological and psychiatric manifestations, including fatigue, depression, and anxiety [29]. The proposed mechanisms involve cytokine-mediated neuroinflammation, endothelial dysfunction, and autoantibody formation, which may further aggravate psychiatric vulnerability in patients with chronic diseases such as hepatitis B. These findings support the notion that chronic viral infection and systemic inflammation act synergistically to influence both mental health and hepatic pathology.

The diagnosis and management of viral hepatitis remain a global health challenge despite advances in screening and antiviral therapy. Recent studies have discussed difficulties in early detection, long-term follow-up, and treatment adherence across different types of hepatitis [30–33]. These publications highlight the ongoing burden of viral hepatitis worldwide and the complexity of comorbid liver diseases that influence clinical outcomes. Although our study did not focus on diagnostic or therapeutic aspects, our findings emphasize an additional layer of complexity in hepatitis B management—the psychological dimension. Addressing psychiatric symptoms such as depression, anxiety, and fatigue in conjunction with hepatic inflammation may provide a more holistic approach to patient care.

The acquisition and persistence of hepatitis B virus (HBV) infection are influenced by multiple demographic, behavioral, and biological factors. Major routes of transmission include perinatal and parenteral exposure, both of which play a crucial role in global HBV epidemiology. In particular, vertical transmission remains one of the most significant risk factors, and the maternal viral load has been identified as a key determinant of intrauterine infection [34]. Although our study focused on adults with established chronic HBV infection, these findings emphasize that persistent viral replication and associated necroinflammatory activity may not only contribute to hepatic disease progression but also indirectly increase psychological distress through chronic immune activation, stigma, and uncertainty regarding disease prognosis.

Taken together, our findings provide evidence that both biological processes (such as necroinflammation and fibrosis) and psychosocial factors (such as age, gender, and marital status) jointly shape the psychological well-being of CHB patients and consistent with qualitative evidence by Freeland et al., who showed that HBV profoundly affects health-related quality of life in U.S. patients [35]. Furthermore, Wu et al. identified a high incidence of sleep disorders among CHB patients, highlighting the broader psychiatric spectrum beyond depression, anxiety, and fatigue [36]. These results emphasize the need for integrated care models that incorporate mental health screening and support into the routine management of CHB. Addressing depression, anxiety, and fatigue in this population may not only improve quality of life but also enhance treatment adherence and potentially influence disease outcomes.

## Strengths and limitations

The strengths of this study include its relatively large sample size, use of validated psychological scales, and comprehensive evaluation of non-invasive fibrosis markers. Nevertheless, several limitations should be acknowledged. First, the cross-sectional design precludes causal inference between liver disease severity and psychological outcomes. Second, potential residual confounding (e.g., unmeasured social or clinical variables) cannot be excluded. Psychosocial factors such as stress level, social support, and perceived stigma may also influence depression and anxiety in patients with chronic diseases; however, these variables were not assessed in the present study. Future studies combining biological, psychological, and social perspectives may provide a more comprehensive understanding of mental health in chronic hepatitis B. Third, self-reported measures may introduce reporting bias. Another limitation of our study is the absence of elastography data, which could have provided a more precise evaluation of hepatic fibrosis. Since transient elastography was not routinely performed for all patients during the study period, fibrosis assessment relied on indirect non-invasive indices (APRI and FIB-4), which have been previously validated as surrogate markers of liver injury and necroinflammation. Absence of histopathological or elastographic fibrosis staging, which prevented formal classification into F1–F4 categories is also another important limitation of our study. Non-invasive indices were therefore used as surrogate markers of hepatic injury and necroinflammation rather than exact fibrosis grades. Future longitudinal studies are warranted to clarify temporal relationships and explore mechanisms underlying these associations.

## Conclusions

This study highlights the multifactorial nature of psychological symptoms—particularly depression, anxiety, and fatigue in patients with chronic hepatitis B. Among non-invasive fibrosis indices, fatigue was significantly associated with higher APRI scores, and anxiety was related to higher API scores, whereas no significant association was observed between depression and fibrosis measures. Female sex and lack of marital support appeared to increase psychological vulnerability, while antiviral treatment was associated with greater fatigue. These findings emphasize that the psychological burden of CHB cannot be explained solely by psychosocial factors but may also reflectbiological processes related to hepatic inflammation and injury.

From a clinical perspective, routine screening for depression, anxiety, and fatigue should be integrated into the management of CHB. Addressing these symptoms through psychosocial support, early psychiatric interventions, and patient-centered care strategies may improve quality of life, enhance treatment adherence, and potentially influence long-term outcomes.

Future longitudinal and mechanistic studies are warranted to clarify the bidirectional relationships between liver disease activity and psychological symptoms, and to determine whether targeted interventions can improve both mental health and hepatic outcomes in CHB patients.

## Data Availability

The datasets used and/or analysed during the current study are available from the corresponding author on reasonable request.

## Declarations

### AI statement

The authors used AI and AI-assisted Technologies in the writing process. These technologies improved the readability and language of the work. Still, they did not replace key authoring tasks such as producing scientific or medical insights, drawing scientific conclusions, or providing clinical recommendations. The authors are ultimately responsible and accountable for the contents of the whole work.

## Abbreviations

HBV: Hepatitis B Virus
CHB: Chronic Hepatitis B
AST: Aspartate aminotransferase
ALT: Alanine aminotransferase
FIB-4: Fibrosis 4 Score
API: Age to platelet index
AAR: AST to ALT ratio
APRI: AST to Platelet ratio index
HADS: Hospital Anxiety and Depression Scale
FSS: Fatigue Severity Scale
PSM: Propensity Score Matching
VIF: Variance Increase Factor
IQR: Inter Quantile Range
OR: Odds ratio
TDF: Tenofovir disoproxyl fumarat
TAF: Tenofovir alafenamide
